# Relationship Between Gait Stability After Total Knee Arthroplasty and Preoperative Physical Function and Gait Variability: A Cross-Sectional Study

**DOI:** 10.7759/cureus.80714

**Published:** 2025-03-17

**Authors:** Kunihiro Onishi, Yasushi Miura, Shigeharu Tanaka, Ryoma Nakatani, Nobuhisa Sato, Hiroyoshi Iwaki, Kazuhiro Matsui

**Affiliations:** 1 Department of Rehabilitation, Osaka Orthopedic Hospital, Osaka, JPN; 2 Department of Rehabilitation Science, Kobe University Graduate School of Health Sciences, Kobe, JPN; 3 Department of Rehabilitation, Faculty of Health Science, Tokyo Kasei University, Sayama, JPN; 4 Department of Rehabilitation, Ishikawa Hospital, Himeji, JPN; 5 Department of Orthopedic Surgery, Tsukazaki Hospital, Himeji, JPN; 6 Department of Orthopedic Surgery, Osaka Orthopedic Hospital, Osaka, JPN; 7 Graduate School of Information Science and Arts, Osaka Electro-Communication University, Osaka, JPN; 8 Graduate School of Engineering Science, The University of Osaka, Osaka, JPN

**Keywords:** accelerometer, gait variability, total knee revision arthroplasty, walking independence, walking patterns

## Abstract

Background

Gait analysis studies have provided valuable insights into gait variability and patterns across various patient populations. However, limited research has examined the relationship between preoperative physical functions, gait variability, and walking independence following total knee arthroplasty (TKA). This study aims to evaluate the potential utility of accelerometers for assessing preoperative gait characteristics by investigating the association between preoperative gait variability, physical functions, and the time required to achieve cane-walking independence after TKA.

Methods

We assessed 68 patients who underwent unilateral TKA. Preoperative physical therapy evaluations included measurements of knee flexion range of motion (ROM), knee extensor strength, walking pain, 10-meter walking speed (10 MWS), and gait coefficient of variation (CV). An acceleration sensor was attached to the spinous process of the third lumbar vertebra during walking to obtain CV results. A correlation analysis was then performed to examine the relationship between preoperative assessments and the number of days required to achieve cane-walking independence after TKA.

Results

The number of days required for cane-walking independence after TKA was negatively correlated with knee flexion ROM (r = -0.264, p < 0.05), knee extensor strength (r = -0.410, p < 0.01), and 10 MWS (r = -0.365, p < 0.01). In contrast, it was positively correlated with gait cycle CV (r = 0.374, p < 0.01).

Discussion

Preoperative knee joint function appears to influence the rehabilitation process following TKA. Furthermore, assessing gait variability quantitatively is essential when evaluating gait stability after TKA and preoperative physical functions.

Conclusions

Accelerometers provide a simple and effective method for quantifying gait characteristics in patients with knee osteoarthritis.

## Introduction

Knee osteoarthritis (KOA) is a degenerative joint disease primarily characterized by pain and commonly affecting around 25 million older adults in developed countries [1]. The condition leads to pain and muscle weakness, which impairs walking ability, restricts daily activities, and potentially increases healthcare costs. In Japan, where the aging population is substantial, healthcare-related social security expenses reached 47.42 trillion yen in 2021 and are expected to keep rising [2]. Therefore, preventing gait deterioration in KOA patients is essential for reducing future healthcare costs, highlighting the critical importance of effective interventions.

For patients with late-stage KOA, total knee arthroplasty (TKA) is recommended to relieve pain, enhance physical function, and improve quality of life [3]. However, Berghmans et al. [1] reported that even one year after surgery, recovery of knee joint muscle strength and walking speed remained at 72.0-91.7% and 87.3-94.2%, respectively, compared to healthy individuals. This indicates that despite the significant financial and physical burden of TKA, some patients continue to experience limitations in their physical functions and walking ability. Therefore, identifying factors contributing to these postoperative residual impairments is essential.

KOA patients often exhibit abnormal gait patterns due to various functional impairments. These abnormalities can be quantitatively assessed using triaxial accelerometers, which offer a noninvasive, user-friendly method for real-time monitoring of gait patterns [4]. Among gait stability indices, the coefficient of variation (CV) of gait cycle time has been proposed as a valuable metric for assessing dynamic gait stability [5]. Hausdorff et al. [6] demonstrated that CV serves as a predictive marker for future falls in a cohort of 52 community-dwelling older adults, outperforming other parameters such as muscle strength, balance ability, and gait speed. These findings suggest that CV is a valuable indicator for assessing gait stability and promoting independent walking.

While previous studies have examined the impact of preoperative muscle strength and pain on postoperative rehabilitation outcomes [7], the relationship between changes in gait cycle time CV and postoperative gait independence remains inadequately explored. The recovery of gait ability after TKA varies significantly among individuals, with some patients failing to achieve sufficient improvement in walking function [8,9]. Notably, early gait recovery during postoperative rehabilitation can reduce fall risk and promote functional restoration [10].

Given this context, the present study investigates the relationship between preoperative gait assessment and postoperative gait recovery to optimize rehabilitation strategies for TKA patients. Preoperative gait assessment is essential for predicting functional recovery and developing appropriate rehabilitation plans after TKA. The CV of gait cycle time, which reflects gait stability, has been identified as a useful predictor of fall risk [6] and rehabilitation progress [4,11,12]. Additionally, previous studies suggest that preoperative knee extensor strength and gait speed play significant roles in achieving independent postoperative ambulation [8]. Assessing these factors postoperatively may help optimize individualized rehabilitation plans tailored to each patient’s needs.

This study aims to evaluate the utility of preoperative assessments by examining the relationship between the number of days required to achieve cane-walking independence after TKA and preoperative physical function and gait variability. By analyzing the association between gait CV, knee joint function, and the time needed to attain cane-walking independence, this research explores the applicability of accelerometer-based gait assessments in the preoperative management of TKA. The findings will contribute to understanding how accelerometer-measured gait variability can aid in risk stratification before TKA and the development of individualized rehabilitation strategies for TKA patients.

## Materials and methods

Study procedure and participants

The study was designed as a prospective cohort study, with data collection conducted from February 2019 to December 2020. The inclusion criteria for participants were (1) patients over 60 years old diagnosed with medial KOA who underwent unilateral TKA and (2) patients capable of walking independently or with the use of a cane before TKA. The exclusion criteria were (1) contralateral TKA; (2) orthopedic or other conditions affecting walking ability; and (3) unavailability of data.

The study was approved by the institutional review boards (IRBs) of the participating institutions before its implementation (Tsukazaki Hospital IRB approval number 261013 and Ishikawa Hospital IRB approval number 2019-1). All patients were thoroughly informed about the study’s objectives and methods and assured that their participation would not result in any disadvantages. Written consent was obtained from all participants prior to their inclusion in the study.

Measurements

This subsection describes the measurements conducted on patients before TKA. Knee joint flexion range of motion (ROM) was assessed during active motion using a plastic angle meter. Knee extensor strength was measured with a handheld dynamometer (µTas F1; ANIMA). Participants were seated with their knees flexed at 90°, hands resting on their thighs. The handheld dynamometer was secured with a belt positioned to contact the proximal 3 cm of each outer malleolus and was anchored to the foot of the bed.

Participants performed two maximal isometric contractions of the knee extensor muscle, each lasting three seconds. Knee extensor muscle strength (Nm/kg) was calculated by multiplying the maximum force value (N) by the leg length (m) and then dividing by body weight (kg). Walking pain was evaluated using a numerical rating scale.

The 10-meter walking speed (10 MWS) was measured over a 10-m section, with a 3-m interval allowed for acceleration and deceleration. The measurement recorded the time taken for the patient’s lower limbs to cross the start and finish lines, performed at their preferred pace. Two measurements were taken, and the faster result was recorded, with values expressed in m/s.

Gait analysis

Patients were asked to walk a 15-m section at a comfortable pace once, using a cane. A triaxial accelerometer (MVP-RF-GC-2000; MicroStone) was attached to the spinous process of the third lumbar vertebra, following the procedure described in a previous study [13]. Calibration was performed before mounting the device, and acceleration data during walking was recorded on a PC via Bluetooth, with a sampling frequency of 200 Hz.

Data analysis was conducted using MATLAB R2017b. The acceleration signals were processed through a 0.1-20 Hz bandpass filter. Initial contact was identified by detecting the peak of the acceleration signal in the anteroposterior direction of the third lumbar vertebra, as outlined in a previous study [13].

To determine which gait cycle was being used, the first step at the start of the gait was recorded as either the surgical or nonsurgical side. The analysis interval was set to 5-10 gait cycles to ensure a stable gait. The CV values were calculated using the formula: CV = (SD of gait cycle time / mean gait cycle time). Measurements were obtained from both the surgical and nonsurgical sides during this interval, and the resulting CV values were averaged for both sides combined [14].

Rehabilitation protocol

The postoperative rehabilitation protocol in this study was standardized, with all patients at both facilities following the same regimen. Specifically, mobilization and walker-assisted walking began on postoperative day 1, depending on the patient's pain tolerance. At the same time, ROM exercises and muscle strength training were initiated. While the rehabilitation program’s progression was adjusted according to each patient's condition, it was generally carried out following a consistent protocol.

Statistical analysis

The relationship between the number of days required for cane-walking independence after TKA and preoperative outcomes was assessed using Spearman’s correlation. A significance level of α = 0.05 was applied for the statistical analysis, which was conducted using IBM SPSS Statistics for Windows, Version 22.0 (Released 2013; IBM Corp., Armonk, NY, USA).

## Results

Patients’ characteristics

We examined 68 patients who underwent unilateral TKA, including 57 females (83.8%) and 11 males (16.2%). The baseline characteristics and demographics of the patients are presented in Table 1. The average age of the participants was 75.5 ± 5.8 years, with a BMI of 26.2 ± 4.4 kg/m². All patients were diagnosed with medial KOA and underwent unilateral TKA.

**Table 1:** Baseline demographics and clinical characteristics of the study population (n = 68) Values are presented as means ± SD or percentages. TKA, total knee arthroplasty

Variable	Mean (SD) or percentage (%)
Age (years)	75.5 (5.8)
Gender (female/male)	83.8/16.2
Height (cm)	152.4 (6.5)
Weight (kg)	60.9 (10.7)
BMI (kg/m²)	26.2 (4.4)
Kellgren-Lawrence grade (Grade II/Grade III/Grade IV)	3/35/30
Operative side femorotibial angle before TKA (°)	182.3 (7.5)
Operative side femorotibial angle after TKA (°)	175.7 (2.7)

Number of days required for walking independence and before TKA outcomes

Table 2 presents the number of days required to achieve walking independence and before TKA outcomes.

**Table 2:** Number of days required for walking independence and before TKA outcomes Values are presented as means ± SD. 10 MWS, 10-meter walking speed; CV, coefficient of variation; NRS, Numerical Rating Scale; ROM, range of motion; TKA, total knee arthroplasty

Variable	Value (mean ± SD)
Number of days required for walking independence (days)	9.9 ± 6.4
Knee flexion ROM on the affected side (°)	119.0 ± 14.1
Knee flexion ROM on the contralateral side (°)	124.3 ± 13.4
Knee extensor strength on the affected side (Nm/kg)	0.82 ± 0.4
Knee extensor strength on the contralateral side (Nm/kg)	0.90 ± 0.4
NRS on the affected side (mm)	3.7 ± 2.3
10 MWS (s)	1.14 ± 0.3
CV (%)	4.30 ± 3.4

Relationship between the number of days required for cane-walking independence and before TKA outcomes

Table 3 presents the relationship between the number of days required for cane-walking independence and before TKA outcomes. The strength of the correlations was classified according to Cohen’s criteria [15]: weak correlation (0.10 ≤ |r| < 0.30), moderate correlation (0.30 ≤ |r| < 0.50), and strong correlation (|r| ≥ 0.50). Knee ROM showed a weak negative correlation (r = -0.264, p < 0.05), while knee extensor strength (r = -0.410, p < 0.01), 10 MWS (r = -0.365, p < 0.01), and the CV of the gait cycle (r = 0.374, p < 0.01) showed moderate correlations.

**Table 3:** Correlation between the number of days required for cane-walking independence and before TKA outcomes 10 MWS, 10-meter walking speed; CV, coefficient of variation; NRS, Numerical Rating Scale; ROM, range of motion; TKA, total knee arthroplasty

Variable	r	p-Value
Age	0.047	0.702
Knee flexion ROM on the affected side	-0.264	p < 0.05
Knee extensor strength on the affected side	-0.41	p < 0.01
NRS on the affected side	-0.103	0.403
10 MWS	-0.365	p < 0.01
CV	0.374	p < 0.01

Additionally, the effect size was evaluated using Cohen’s d, interpreted based on the standard classification: small effect (d ≥ 0.2 and < 0.5), medium effect (d ≥ 0.5 and < 0.8), and large effect (d ≥ 0.8). These findings indicate that preoperative knee extensor strength and gait variability may significantly influence the time required to achieve cane-walking independence.

## Discussion

In this two-center study, we examined the relationship between preoperative factors and the number of days required to achieve cane-walking independence after TKA. We initially hypothesized that knee extensor strength and CV would be strongly associated with the time needed for cane-walking independence. Our findings showed a positive correlation between CV during walking and the number of days required for cane-walking independence. Conversely, negative correlations were observed with preoperative knee flexion ROM, knee extensor strength, and 10 MWS. These results suggest that both preoperative physical function and gait variability are important factors to consider when assessing the time required to regain cane-walking independence following TKA.

This study underscores the importance of preoperative gait analysis in the rehabilitation process of patients undergoing TKA. Previous research by Christensen et al. [16] demonstrated that preoperative knee extension strength plays a significant role in postoperative walking ability and daily activities. Furthermore, they reported a reduction in quadriceps strength and knee extension moment during walking on the operated side even six months after TKA [17], emphasizing the need for quadriceps strength recovery to enhance postoperative walking ability. Gait pattern changes are often linked to aging and diminished knee extensor strength. Park et al. [18] found a 40% decrease in knee extension strength among KOA patients compared to age-matched individuals without KOA, highlighting its potential impact on walking. Additionally, studies by Hausdorff et al. [6] involving community-dwelling elderly individuals and Spinoso et al. [19] involving KOA patients support the association between gait patterns and reduced knee extension strength, which aligns with our findings.

In contrast, Hiyama et al. [11] reported that, although knee ROM, knee extensor strength, and walking speed declined after TKA, there were no significant changes in gait CV before and after surgery, with stable gait achieved by discharge. They suggested that even patients with reduced physical function after TKA maintained consistent gait CV, which contrasts with the findings of our study. Notably, the patients in Hiyama et al. [11] were younger and had greater bilateral knee extensor strength compared to those in our study, which may explain the differing results.

No specific studies have directly investigated the relationship between preoperative walking speed and the number of days required to achieve independent walking after TKA. While previous research has established links between preoperative gait function and postoperative outcomes in TKA patients, the connection between preoperative gait function and the time required for cane-walking independence remains unexplored [11]. Hiyama et al. [11] calculated the CV of gait in TKA patients but did not examine its association with the number of days needed to achieve cane-walking independence.

Furthermore, there is still no clear consensus on the direct relationship between the time required for cane-walking independence and postoperative walking speed. A meta-analysis by Abbasi-Bafghi et al. [20] reported a significant improvement in walking speed within the six to 60-month period following TKA; however, the factors contributing to this improvement, including the role of achieving cane-walking independence, remain unclear. While our study provides new insights, further research is needed to address the limited studies available on this topic.

The findings of this study indicate that the CV of the gait cycle, measured using an accelerometer, is significantly associated with the number of days required to achieve cane-walking independence after TKA. This suggests that assessing gait stability preoperatively through CV measurement can be valuable for risk stratification.

Traditionally, preoperative evaluations for TKA have focused on knee ROM, knee extensor strength, and walking speed [8,9]. However, these parameters may not always accurately predict postoperative gait recovery. In contrast, assessing gait variability using an accelerometer offers a quantitative measure of gait stability, providing valuable insights for evaluating fall risk and developing effective rehabilitation strategies.

Falls among older adults can have serious consequences, making their prevention and prognosis essential. Chang et al. [21] reported that asymmetric gait patterns, postural instability, and increased forward trunk displacement are linked to reduced balance and a higher risk of falls during the early postoperative period, especially among hospitalized TKA patients. This emphasizes the importance of fall prevention during hospitalization from a risk management perspective.

Patients with higher CV values tend to exhibit more significant gait rhythm disturbances, which can delay their achievement of cane-walking independence. Implementing preoperative balance training and gait rehabilitation for these patients may be beneficial. Additionally, stratifying patients based on preoperative CV values and introducing targeted fall prevention programs for high-risk groups could help lower postoperative fall risk and reduce hospitalization periods.

Considering the clinical application of accelerometers, ease of use is a critical factor. This study confirmed that, compared to traditional gait assessment methods like gait speed measurement and motion analysis systems, accelerometers offer the advantage of quickly and conveniently acquiring gait data. Their simplicity and noninvasiveness make them promising as practical screening tools in clinical settings.

The increasing availability of wearable sensors has also made it possible to assess gait outside of hospital environments, which could enhance preoperative rehabilitation by enabling remote monitoring. However, this study did not directly evaluate the potential burden related to measurement duration or data processing, which remains an area for future research.

To further establish the practicality of accelerometers, additional validation studies in clinical settings and assessments of usability by healthcare professionals are essential.

Limitations

This study has several limitations. First, the Kellgren-Lawrence grade of the non-operated knee was not assessed. Asymmetry in quadriceps strength after TKA is associated with asymmetrical knee joint movement [22] and increased load on the non-operated side, highlighting the importance of evaluating the contralateral knee structure [16,23].

Second, balance function was not assessed. Pua et al. [24] emphasized that balance is a critical factor when prescribing walking aids due to its relation to postural sway in the frontal plane, indicating that a standing balance assessment should have been included.

Third, psychological factors were not considered. Previous studies have shown that anxiety and depression are related to pain, while age and gender are specifically associated with gait function [25]. Additionally, factors like the type and dosage of analgesics can influence psychological conditions [26], which were not examined in this study.

Fourth, the influence of preoperative use of assistive devices and the need for individualized gait function analysis should be addressed. Patients who used a cane preoperatively may have different baseline gait abilities, potentially affecting CV measurements. Therefore, stratifying patients into non-cane and cane-user groups and examining differences in their gait recovery processes would be necessary.

Fifth, while this study demonstrated the potential utility of accelerometer-based gait assessment for preoperative risk evaluation and rehabilitation planning in TKA patients, its application may be limited to certain medical institutions. Accessibility remains a challenge, especially in resource-limited healthcare settings.

Regarding future research, although this study established a connection between preoperative gait rhythm CV and postoperative gait independence, its contribution to predicting actual fall risk and long-term gait function remains unclear due to insufficient data collection. Further investigation is needed to address this gap.

Additionally, this study focused on a specific patient population and did not consider potential interactions with other preoperative factors, such as psychological conditions or pain management. Future research should include a more diverse patient cohort to explore how CV can be effectively integrated into postoperative rehabilitation strategies to optimize functional recovery.

## Conclusions

We examined the relationship between gait stability and preoperative physical function in TKA patients and found that assessing preoperative physical functions and gait rhythm can effectively predict the time required for achieving cane-assisted walking after TKA. Using accelerometers offers a convenient way to quantify gait characteristics in KOA patients.
